# Oxidative Stress and Cortical Thickness in the Psychosis Spectrum: Investigating Glutathione Metabolism and Antioxidant Defenses in Youth

**DOI:** 10.1192/j.eurpsy.2025.1164

**Published:** 2025-08-26

**Authors:** R. Moreno-Comellas, M. Ortuño, I. Martínez, F. Fesce, E. de la Serna, J. Castro-Fornieles, I. Baeza, G. Sugranyes

**Affiliations:** 1 Psychiatry, Benito Menni CASM; 2 Department of Child and Adolescent Psychiatry and Psychology; 3 Psychiatry, Institute of Neuroscience, University of Barcelona, Barcelona; 4 CIBERSAM, Madrid, Spain; 5 Psychiatry, CIBERSAM, Madrid, Spain

## Abstract

**Introduction:**

Oxidative stress, particularly through disruptions in glutathione metabolism and Total Antioxidant Capacity (TAC), has been implicated in cortical thinning and other brain structural changes seen in psychosis. These changes may be more pronounced in the early psychosis spectrum, but this relationship remains underexplored.

**Objectives:**

This study investigated the relationships between key oxidative stress markers—reduced glutathione (GSH), oxidized glutathione (GSSG), the GSH/GSSG ratio, and TAC—and cortical thickness in the cingulate, insula, and fronto-temporal brain regions.

**Methods:**

A total of 57 youths on the early psychosis spectrum and 44 healthy controls participated, with a mean age of 15.51 years. There were no significant differences in age or sex between the groups. Cortical thickness was measured using MRI, and blood samples were analyzed for oxidative stress markers. Partial correlations were performed, controlling for total intracranial volume, age, and sex, to examine the relationships between oxidative stress markers and cortical thickness. A permutation analysis was then conducted to assess group differences in these associations.

**Results:**

In healthy individuals, a higher GSH/GSSG ratio was significantly associated with increased cortical thickness in the right insula (r = 0.50, p < 0.05). Conversely, in the early psychosis spectrum group, there was a consistent trend of negative correlations between TAC and cortical thickness, particularly in the left frontal cortex. Permutation analysis revealed significant group differences in the association between GSH and cortical thickness in both the left insula and left temporal regions (p < 0.05). Additionally, TAC showed significant differences in its relationship with cortical thickness in the left and right frontal regions (p = 0.01 and p < 0.05), indicating asymmetrical oxidative stress involvement across hemispheres in the early psychosis spectrum group compared to healthy controls.

**Conclusions:**

These findings suggest that oxidative stress markers, particularly related to glutathione metabolism and TAC, are linked to regional cortical thickness variations. The results highlight distinct oxidative stress effects in the early psychosis spectrum compared to healthy individuals, emphasizing the potential role of oxidative stress markers as early indicators of neuroanatomical changes in psychosis.

**Disclosure of Interest:**

R. Moreno-Comellas: None Declared, M. Ortuño: None Declared, I. Martínez: None Declared, F. Fesce: None Declared, E. de la Serna: None Declared, J. Castro-Fornieles: None Declared, I. Baeza: None Declared, G. Sugranyes Grant / Research support from: This study has been funded by the Spanish Ministry of Health, Instituto de Salud Carlos III «Health Research Fund»/ FEDER funds (PI2100330, FORT23/00002_SUGR_G6) and La Marató TV3 (grant number 202232-30-31).

